# Widespread peat carbon losses driven by the 2025 Scottish megafire

**DOI:** 10.1038/s41561-026-01994-3

**Published:** 2026-05-20

**Authors:** Johanna Schoenecker, Martin J. Baur, Juliana Kohli, Sarah Baker, Matthew Jones, Sander Veraverbeke, Alexandra G. Konings, Adam F. A. Pellegrini

**Affiliations:** 1 Department of Plant Sciences, University of Cambridge, Cambridge, UK; 2 WildFIRE Lab, University of Exeter, Exeter, UK; 3 Tyndall Centre for Climate Change Research, School of Environmental Sciences, University of East Anglia, Norwich, UK; 4 Faculty of Science, Vrije Universiteit Amsterdam, Amsterdam, The Netherlands; 5 Department of Earth System Science, Doerr School of Sustainability, Stanford University, Stanford, CA USA

**Keywords:** Natural hazards, Environmental impact, Carbon cycle, Fire ecology

## Abstract

Drier and warmer climates have allowed fires to increasingly burn carbon-dense peatland ecosystems. Here we document a 2025 Scottish megafire in the UK, which spread rapidly and burned severely across peatlands in Scotland with anomalously low soil moisture, emitting 38,600 MgC (25,200–119,000 MgC). Peat combustion contributed nearly 85% of total emissions, suggesting drier climates increase fire emissions from peat, which can require decades to centuries to recover.

## Abstract (web summary)

In summer 2025, a rapidly spreading megafire in Scotland, twice as large as the next-largest UK fire in the past two decades, caused widespread carbon emissions from the combustion of vegetation and peat, according to remote sensing analyses, field measurements and modelling.

## Main

Fires are becoming larger and more severe in many ecosystems worldwide, resulting in increasing ecosystem impacts and carbon emissions^1,2^. Changing wildfire regimes in peatlands, which have rarely burned historically, are especially concerning. When peatlands do burn, they can produce large carbon emissions through the combustion of organic soil^3,4^ (for example, 78% of total fire emissions in northern systems^5^) and require centuries to millennia to re-sequester carbon in organic soils^6^. The multicentury recovery of carbon stocks in peat means that emissions can be considered ‘irrecoverable’^7^ from the standpoint of climate mitigation initiatives, which typically focus on achieving net zero during the current century^8^.

High soil moisture in peatlands, sustained even during climatological dry seasons, protects belowground carbon stocks from burning most of the time^1^. In recent decades, tropical peatlands have experienced severe peat-combusting fires, driven by agricultural drainage and occurrence of El Niños leading to low soil moisture^2,3^, while northern peatlands have been less affected. However, warmer and drier climates in northern peatlands^9^ are increasing the occurrence of wildfire-conducive conditions^10^, leading to greater emissions from peat^4^. Here we document the occurrence of the UK’s largest wildfire in at least the last 20 years and argue that it points towards a shift in the UK’s fire regime, with implications for other temperate and northern peatlands.

**Extended Data Fig. 1:**
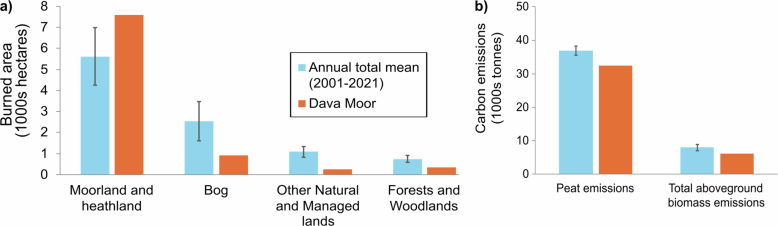
Comparison of Dava Moor Fire burned area and carbon emissions with UK totals (2001 – 2021). **a**) burned area in thousands of hectares split by vegetation type. **b**) carbon emissions in thousands of tonnes split by peat and aboveground biomass emissions. In both panels the bars represent the means and the error bars represent the standard error of the annual emissions across the 2001-2021 period (n = 21). Vegetation type categories and burned areas from 2001-2021 are described in the *Methods*.

The Dava Moor fire formed by the merger of two independently ignited fires at Dava and Carrbridge, northern Scotland, between 28 June and 1 July 2025, and burned approximately 10,000 hectares, making it the UK’s first megafire under a common size-threshold definition^11^. The fire was roughly twice the size of the next-largest UK fire in the past 20 years (the 2019 Flow Country fire) and comparable to the historical average of the UK’s total annual burned area^6^ (2001–2021; Methods; Extended Data Fig. 1). The wildfire spread rapidly and burned severely, burning for just 4 days and over 79% of the burned area classified as high severity, corresponding to near total combustion of aboveground vegetation and probably burning of peat (Methods). In the UK, a country where fires are usually small, often managed and not severe^12^, the scale of this wildfire is unprecedented.

**Fig. 1:**
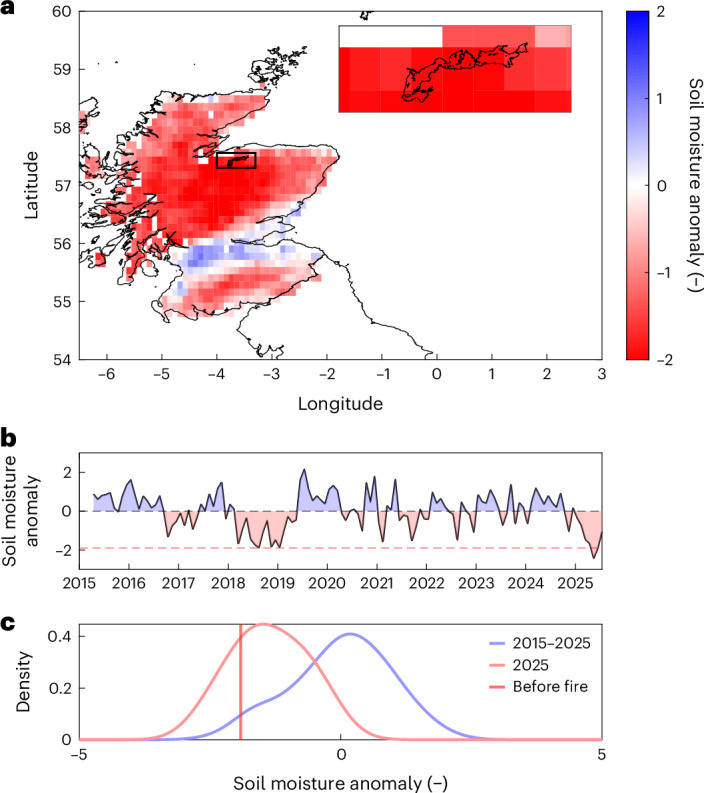
Exceptionally dry conditions in 2025 primed the Dava Moor fire. **a**, Map of the average soil moisture anomalies for June 2025, with an inlay of the Dava Moor fire perimeter. Soil moisture anomalies are calculated relative to respective monthly averages and standard deviations of the entire observational period in a specific grid cell (Methods). **b**,**c**, A focus on pixels within or intersecting the Dava fire area: the time series of average monthly soil moisture anomalies for the Dava Moor fire area (**b**) and the calculated soil moisture anomaly probability density functions for all observations and 2025 observations within the Dava fire area (**c**). The average anomaly for June 2025 in the Dava Moor fire area is displayed as a red line. Basemap data in **a** from GADM v.4.1 (https://gadm.org/).

**Extended Data Fig. 2:**
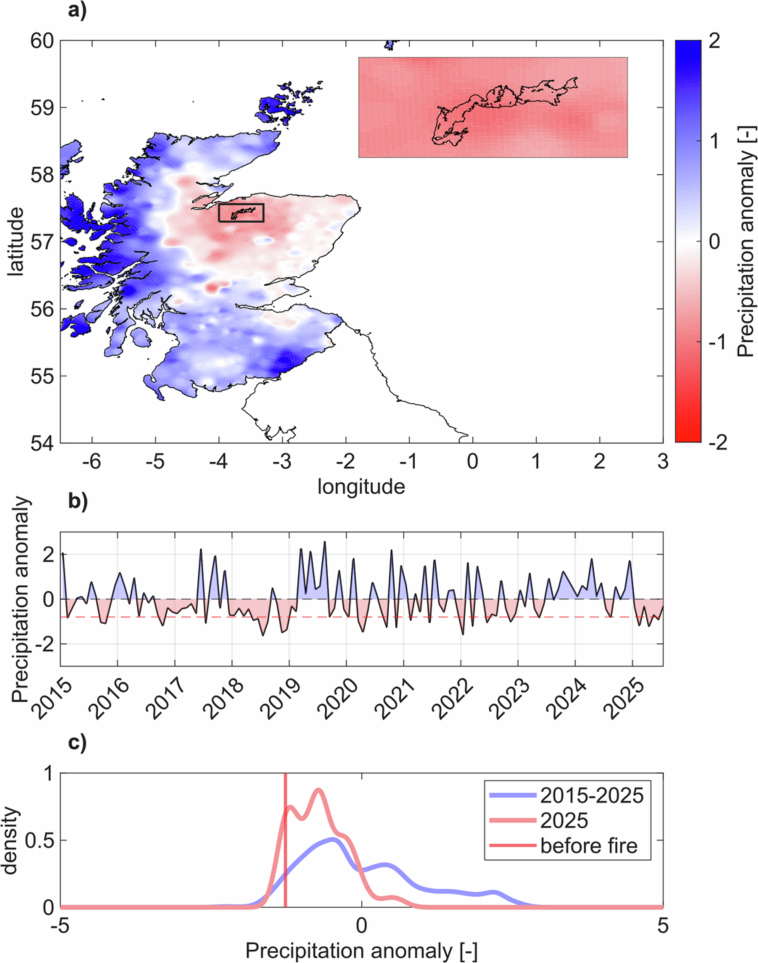
Precipitation anomalies for the Dava Moor Fire area in June 2025 compared with the long-term anomaly distribution. **a**) Map of the average precipitation anomalies for June 2025, with an inlay of the Dava Moor fire perimeter. Precipitation anomalies are calculated relative to respective monthly averages and standard deviations of the entire observational period (*Methods*). **b**) Time series of average monthly precipitation anomalies for the Dava fire area. **c**) Estimated precipitation anomaly probability density functions for all observations and 2025 observations. The average anomaly for June 2025 is displayed as a red line. Basemap data in **a** from GADM version 4.1 (https://gadm.org).

**Extended Data Fig. 3:**
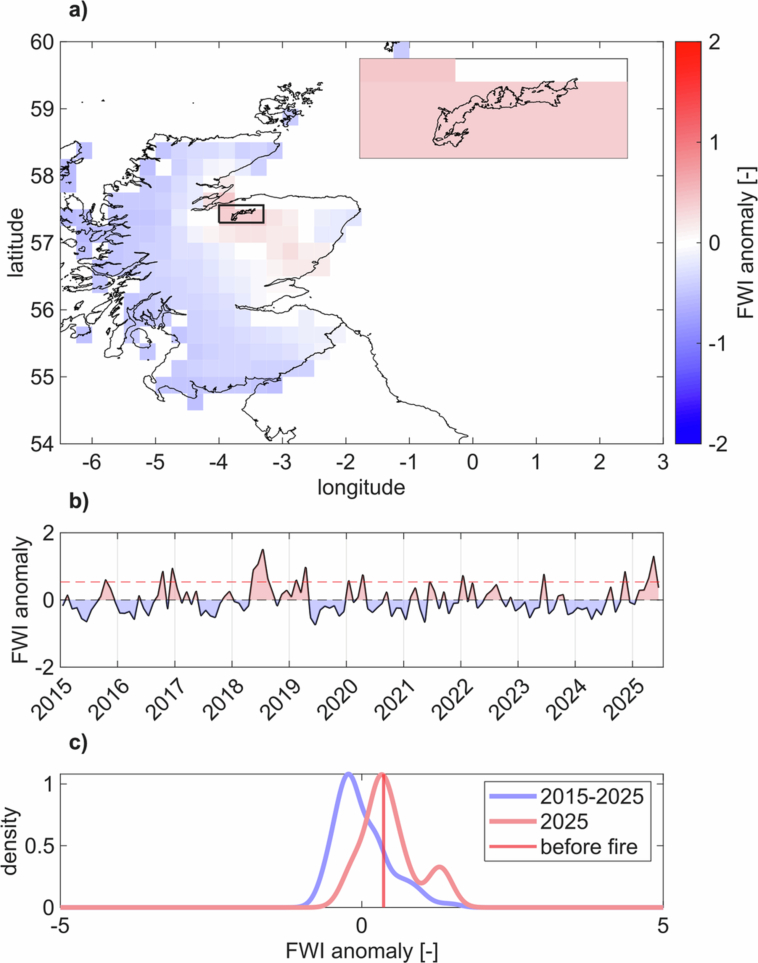
Fire Weather Index anomalies for the Dava Moor Fire area in June 2025 compared with the long-term anomaly distribution. **a**) Map of the average Fire Weather Index (FWI) anomalies for June 2025, with an inlay of the Dava Moor fire perimeter. FWI anomalies are calculated relative to respective monthly averages and standard deviations of the entire observational period (*Methods*). **b**) Time series of average monthly FWI anomalies for the Dava fire area. **c**) Estimated FWI anomaly probability density functions for all observations and 2025 observations. The average anomaly for June 2025 is displayed as a red line. Basemap data in **a** from GADM version 4.1 (https://gadm.org).

**Extended Data Fig. 4:**
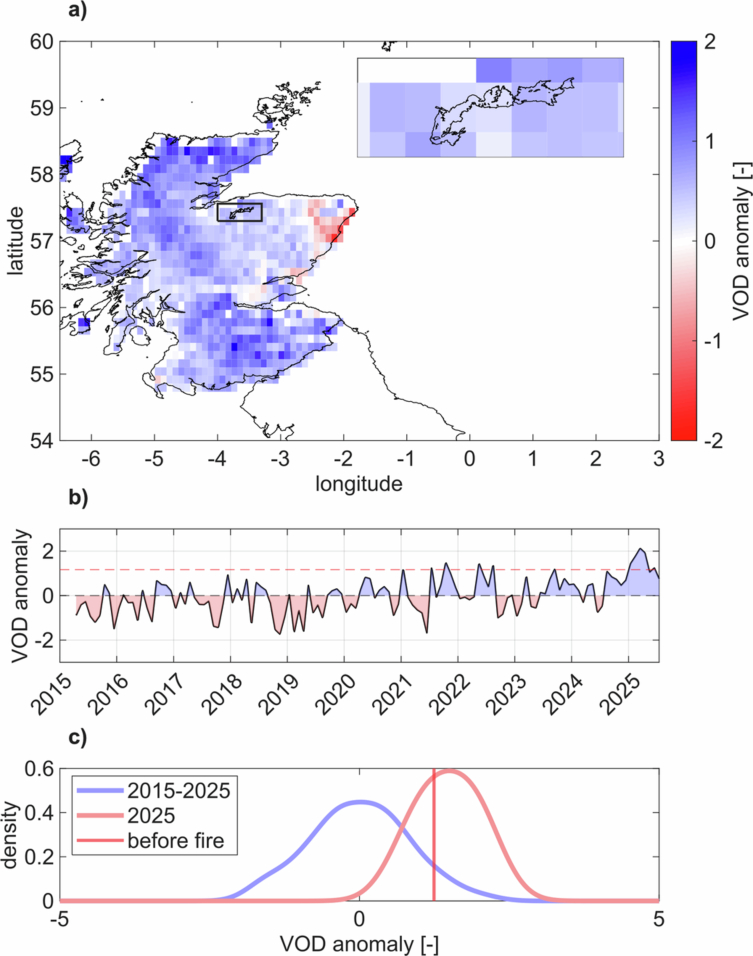
Vegetation optical depth anomalies for the Dava Moor Fire area in June 2025 compared with the long-term anomaly distribution. **a**) Map of the average vegetation optical depth (VOD) anomalies for June 2025, with an inlay of the Dava Moor fire perimeter. VOD anomalies are calculated relative to respective monthly averages and standard deviations of the entire observational period (*Methods*). **b**) Time series of average monthly VOD anomalies for the Dava fire area. **c**) Estimated VOD anomaly probability density functions for all observations and 2025 observations. The average anomaly for June 2025 is displayed as a red line. Basemap data in **a** from GADM version 4.1 (https://gadm.org).

The wildfire occurred under very dry climate conditions. Soil moisture observations by the Soil Moisture Active Passive (SMAP) satellite (Methods; Fig. 1a,b) illustrate that much of Scotland was experiencing unusually dry conditions preceding the fire. At the time and location of the fire, soil moisture was 1.9 s.d. below the decadal monthly average for that location (Fig. 1c). The low soil moisture at the time of the fire is consistent with dry conditions throughout 2025 following a relatively low-precipitation 2024–2025 winter (Extended Data Fig. 2). Meteorological conditions, assessed via the Fire Weather Index (FWI), illustrated that the wildfire location experienced higher FWI values than other areas in Scotland, and that the anomaly was substantially higher in May and marginally higher in June (1.3 and 0.4 s.d. above decadal average, respectively; Extended Data Fig. 3). Concurrently, estimated aboveground fuel load per unit area was 1.2 s.d. above the decadal average for June (estimated via vegetation optical depth^10^; Extended Data Fig. 4). Thus, fuel accumulation probably contributed to the rapid spread, but the dry climatic conditions primed the system to burn.

**Fig. 2:**
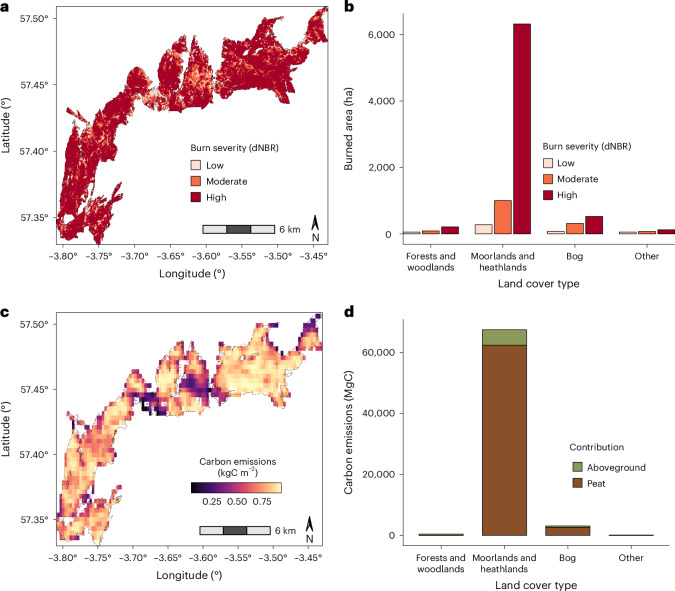
The wildfire was high severity and peat combustion dominated emissions. **a**, The fire severity calculated by the dNBR from Sentinel-2 satellite imagery at 20-m resolution (Methods). **b**, The total burned area partitioned by land cover classes and burn severities using a 2023 land cover map at 10-m resolution. **c**, The total carbon emissions (aboveground and peat) based on the median observed peat burn depth in the respective land cover classes from 106 field measurements and field-measured bulk density values. **d**, The carbon emissions aboveground plant biomass and belowground peat partitioned by land cover classes. All belowground emissions are combustion of peat. Basemap data in **a** and **c** from Sentinel-2, European Space Agency.

Nearly 80% of the total wildfire area was classified as severely burned (determined via the differenced normalized burn ratio (dNBR); Methods; Fig. 2a and Extended Data Table 1). Overlaying the fire data with a 2023 land cover map^13^, we partitioned burn severity among ecosystem types (Methods; Fig. 2b). Moorlands and heathlands, which are shrubby systems, dominated the burned area (83% of burned area; Fig. 2b and Extended Data Table 1). Bogs accounted for 10% of burned area (Fig. 2b and Extended Data Table 1; existing classifications define bogs as areas where peat depths exceed 0.50 m (ref. ^13^)), while forests and other vegetation types comprised 6% of burned area. Even in typically wet bogs, 58% of the fire area burned at high severity (Fig. 2b and Extended Data Table 1).

**Extended Data Fig. 5:**
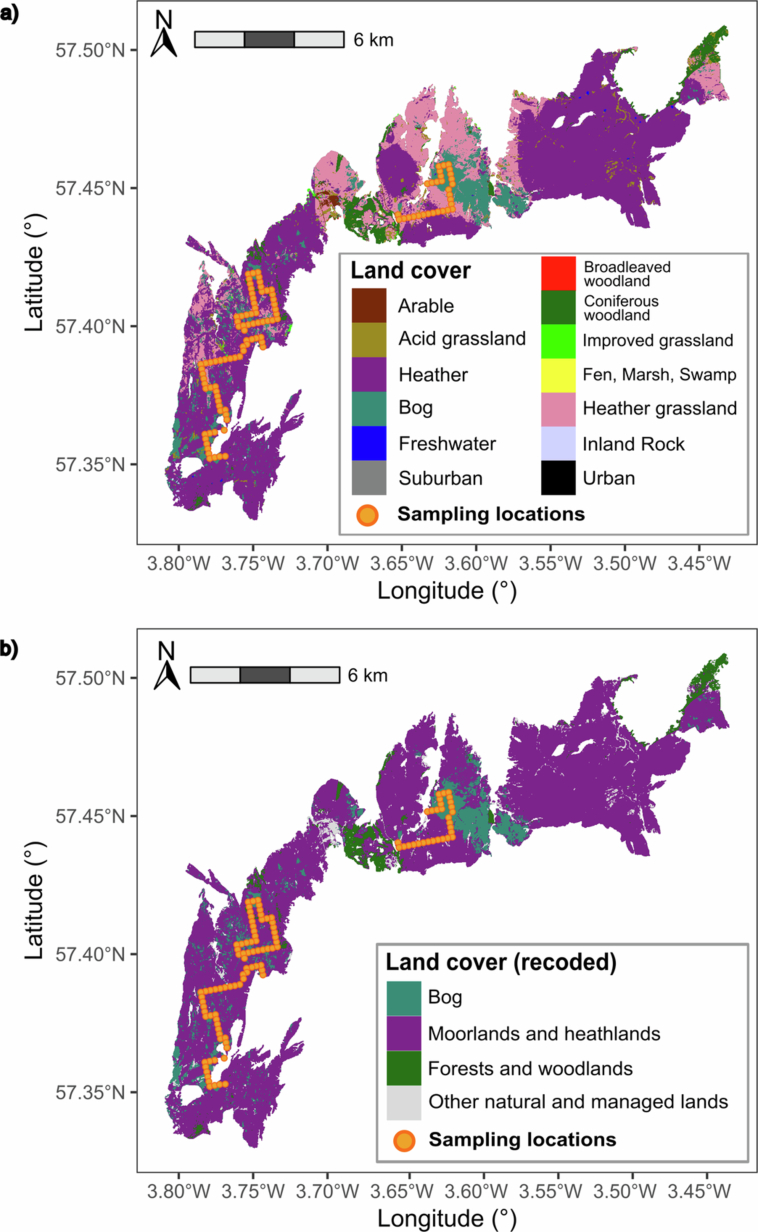
Land cover classification and grouped vegetation types within the Dava Moor fire perimeter. **a**) 2023 UKCEH land cover types^13^ within the fire perimeter at 10 m spatial resolution (*Methods*). **b**) Grouped land cover classes used for emissions calculations. In both maps, locations of field-sampled burn depths are marked in orange. Basemap data in **a** from GADM version 4.1 (https://gadm.org).

**Extended Data Fig. 6:**
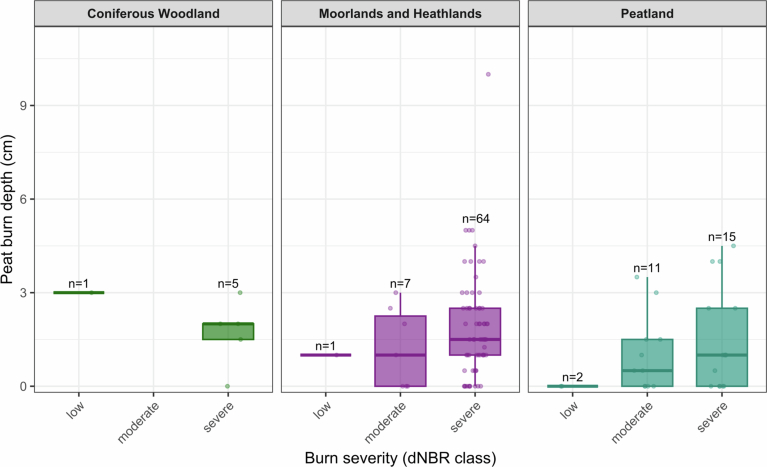
Field-measured peat burn depths by vegetation type and burn severity class. Field-measured peat burn depths (cm; sample size n = 106) within the grouped vegetation types used for emissions calculations (Extended Data Fig. 5b), by burn severity class (based on differenced Normalized Burn Ratio- dNBR). Centre lines show the respective median group value, with boxes representing the interquartile range (25^th^ - 75^th^ percentiles). Whiskers extend to the most extreme values within 1.5 times the interquartile range. Individual data points are overlaid.

We quantified carbon emissions using a bottom-up emissions calculation based on soil moisture, burn depth, carbon density and combustibility of different land cover classes^13^ (Methods). We constrained burn depth with 106 field measurements ca. 1-month post fire and measured peat bulk density in a nearby unburned bog and heathland (Methods; Extended Data Figs. 5 and 6). We also compared our locally optimized bottom-up estimates with those from a global top-down fire emissions product based on observations of fire radiative power emitted from burned areas (the Global Fire Assimilation System; GFAS^12^) and to a an existing UK-specific peat combustion model from ref. ^6^ (referred to here as the Baker Peat Combustion Model).

In this single fire, carbon emissions were 38,600 MgC (25,200–119,000 MgC based on the 25th and 75th quantiles of measured burn depths, as well as 50% and 100% combustion completeness (CC) assumptions; Fig. 2c,d and Extended Data Table 2). Most emissions arose from combustion of peat (84% of total emissions, 32,500 MgC; Fig. 2d and Extended Data Table 2). Moorlands and heathlands, which contributed the most to burned area, also contributed the most to emissions. A large fraction of total emissions from moorlands and heathlands arose from belowground peat combustion, whereas the emissions from the combustion of aboveground vegetation were relatively small (Fig. 2d and Extended Data Table 2). Bogs emitted 1,780 MgC (73% coming from peat combustion; Fig. 2d and Extended Data Table 2). Combustion of peat was the predominant loss pathway in this fire and even occurred in historically wet bogs due to the low soil moisture.

Compared with our locally optimized emissions estimates, the Baker Peat Combustion Model overestimated peat burn depths and bulk density (Extended Data Fig. 6 and Extended Data Table 3; Methods). However, because the Baker Peat Combustion Model did not quantify belowground emissions from non-bog areas, the total fire-wide emissions were comparable to our emissions using median field burn depths and bulk densities (33,000 MgC versus 38,600 MgC, respectively). GFAS-estimated emissions based on fire radiative power were 30,000 MgC. Thus, the GFAS and Baker Peat Combustion Model estimates are within the bounded uncertainty of our locally optimized emissions estimate but tend to be lower (by 14.5% and 23.3%, respectively).

Many lines of evidence point to the 2025 summer climate conditions being more likely in the present climate than in a pre-industrial climate, and that similar conditions will be more common in the future. First, human-caused climate change has increased the risk of wildfire in the UK (for example, sixfold in 2022 ref. ^10^). Second, the rising FWI, especially in the summer, appears to be a trend both locally and regionally^14^, with FWI expected to rise by 24% in Europe by 2050 even under low-emission scenarios^14^. Finally, the UK is projected to experience drier and hotter summers and more droughts (for example, droughts once every 20 years to once every 3 years by 2040 ref. ^14^), which enables peat burning^1^. Consequently, fires are likely to not only grow larger but also burn more severely into peat both in the UK and globally.

**Extended Data Fig. 7:**
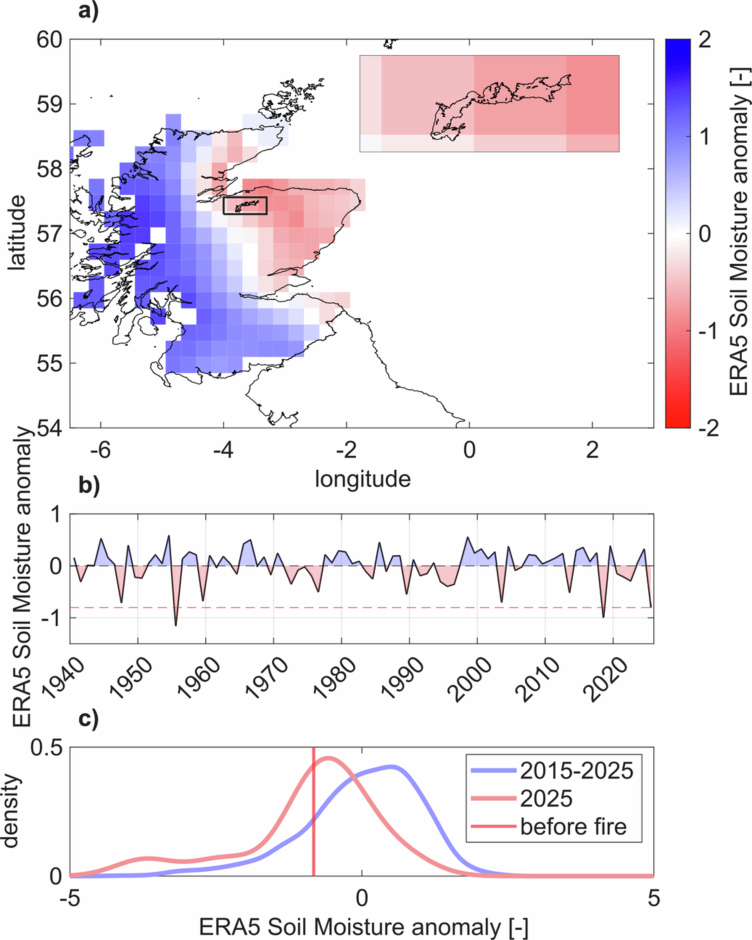
Soil moisture anomalies for the Dava Moor Fire area in June 2025 relative to the 1940-2025 ERA5 record. Soil moisture anomalies using ERA5 reanalysis from 1940-June 2025. The main difference between this figure and the other climate figures is the distribution to calculate the anomaly being based on data from 1940-onwards. **a**) Map of the average soil moisture anomaly for June 2025, with an inlay of the Dava Moor fire perimeter. **b**) Time series of average monthly soil moisture anomaly for the Dava fire area. **c**) Estimated soil moisture anomaly probability density functions for all observations and 2025 observations. The average for June 2025 is displayed as a red line. Basemap data in **a** from GADM version 4.1 (https://gadm.org).

Past drainage of peatlands is common in the UK, which may predispose these peatlands to climate conditions that dry the soil^15^. Although we could not identify peatlands with histories of drainage in the Dava Moor fire area, the soil moisture anomalies are computed relative to the local conditions, putatively capturing effects of potential previous management on the underlying soil moisture (Extended Data Fig. 7). Nonetheless, whether drainage has primed the system to be more sensitive to climate deserves future study.

Although emissions from Dava Moor are small relative to total annual emissions in the UK (for example, 2024 UK agricultural CO_2_ emissions were 1,561,320 MgC (ref. ^16^), 11-fold higher than the emissions from Dava Moor), the emissions were large relative to past UK fires. For example, carbon emissions from Dava Moor were equivalent to 85% of the total mean annual fire emissions from 2001–2021 (38,600 versus 44,900 MgC) of the UK. Consequently, peatland conservation and protection from fire are policy priorities, with fire management measures implemented nationwide to safeguard peatlands. UK policy focuses on restoring peatlands (for example, invested £50 M to restore 35k hectares in 2023), which could help reduce the risk of peat burning during fires.

We propose that these findings highlight that the UK is experiencing extreme wildfire conditions that make peatlands especially vulnerable and give insight into how temperate and northern peatlands may change with global warming. Much of the UK peatland area has experienced extensive land use, which could predispose the peat to drying in these extreme climate events. However, the rising occurrence of hot and dry summers has drastically changed the historically wet climate conditions of many temperate and northern peatlands^17^, resulting in extensive wildfires and carbon emissions^18,19^. Comparison between our field-constrained estimates and existing models for quantifying emissions demonstrated the importance of accounting for local burn depths and peat densities. The presence of highly flammable common heathland species probably contributed to the high severity^20^. Our findings demonstrate that as soil moisture declines, peat losses can rapidly become the dominant source of carbon emissions in wildfires in regions with peaty soils, requiring longer periods of time to recover in an era where fires are increasingly frequent.

## Methods

### Climate conditions and fuel load

To assess the climatic and environmental conditions around the Dava Moor fire, we used soil moisture and vegetation optical depth (VOD) from NASA’s SMAP mission, which uses observations in the microwave spectrum to track water in soils and vegetation^10,21^. SMAP soil moisture has been successfully used to predict burned area in peatlands and to track fire across ecosystems^3,22^. VOD is strongly related to vegetation water content and can give information on fuel load and fuel moisture^23^. Here we used SMAP soil moisture from the baseline single-channel algorithm, while VOD was taken from the dual-channel algorithm, both of which are included in the SMAP Enhanced L2 Radiometer Half-Orbit 9-km EASE-Grid Soil Moisture product, v.6 (ref. ^10^). Soil moisture and VOD are mapped at least every 3 days and at a 9-km spatial resolution. For our analyses, we converted soil moisture and VOD to monthly anomalies relative to the 2015–2025 long-term average for each pixel. To further assess the dry conditions underlying the fire, we also used monthly precipitation estimates from the Met Office’s HadUK-Grid^24^ gridded and regional average climate observations dataset at 1-km spatial resolution, which we also converted to monthly anomalies (accessed 19 September 2025).

The FWI we analysed here is the Canadian Forest Service Fire Weather Index Rating System^9^, which was downloaded from https://ewds.climate.copernicus.eu/datasets/cems-fire-historical-v1?tab=download (accessed 19 September 2025).

To assess how the calculations of anomalies via SMAP may compare with longer-term data, we analysed monthly ERA5 soil moisture data from 1940 to June 2025 (ref. ^25^). We found largely consistent trends around the Dava Moor fire, where 2025 was an anomalously dry year with an especially dry spring (Extended Data Fig. 7).

### Characterizing fire behaviour, intensity and severity

Using fire radiative power (VIIRS, 375-m resolution and MODIS, 1,000-m resolution), we found that the fire front evolved within the southern and northeastern area, tending to transition from high intensity to low intensity at the timescale of days. While fire intensity is used to quantify the energy released through the combustion of biomass in a fire, burn severity as a metric seeks to capture the effects of fire on vegetation and soil^26^.

We obtained the fire perimeter from the European Forest Fire Information System and calculated burn severity in Google Earth Engine, using pre- and post-fire Sentinel-2 surface reflectance imagery (20-m spatial resolution). Both dNBR and relative dNBR (two alternative burn severity metrics), exhibited consistent spatial patterns across the burned area. We therefore used dNBR for subsequent analyses, based on its precedence in Scottish peatland fire studies (for example, refs. ^27–29^). dNBR was calculated as the difference between pre-and post-fire NBR, which is defined as the normalized difference between near-infrared (NIR) and shortwave-infrared (SWIR) reflectance. NBR was derived from cloud-free median composites of 24 pre-fire images (15 May to 24 June 2025) and 10 post-fire images (25 July to 10 August 2025). Burn severity was classified following ref. ^30^ as unchanged (<0.100), low (0.100–0.269), moderate (0.270–0.439) and high (>0.439). dNBR represents burn severity as the magnitude of spectral change between pre- and post-fire conditions. The underlying NBR captures spectral responses of vegetation chlorophyll content, moisture and combustion residue on the exposed soil surface^31^, expressed through contrasting responses in the NIR and SWIR bands, which are high (NIR) and low (SWIR) in green, healthy vegetation and reversed following burning^32^. High severity generally is the complete loss of aboveground vegetation biomass.

### Land cover classifications and field sampling

We assigned different land cover classes to the burned area by overlaying the fire footprint with the land cover map from the UK Centre for Ecology and Hydrology (2023 data, 10-m pixels) and grouped them into ‘bogs’, ‘moorlands and heathlands’, ‘forests and woodlands’ and ‘other natural’. Specifically, we grouped broadleaved woodland and coniferous woodland into ‘forests and woodlands’. We further grouped ‘heather’ and ‘heather grassland’ into ‘moorlands and heathlands’ and all remaining classes were aggregated into ‘other natural and managed lands’. Extended Data Fig. 5 displays the spatial distribution of the two categorizations. Moorland and heathlands are characterized by shrubby species such as heather (*Calluna vulgaris*, *Erica cinerea* and *Erica tetralix*) and gorse (*Ulex* spp.). Note that the Baker Peat Combustion Model calls the ‘bogs’ category ‘peatlands’, which we do not do in our model because of the presence of peat in non-bog land cover classes.

We conducted a field sampling campaign in September 2025 (at approximately 2 months post-fire) to measure peat burn depth across the different land cover types within the burn scar. This helped (1) constrain estimates of burn depths because of the high uncertainties in modelled data and (2) confirm the presence of peat we assumed based on land cover. At 106 locations within the burn scar (on a 200-m evenly spaced point grid), we measured maximum and minimum burn depth in a 1-m^2^ quadrat and assigned the mean of both measurements as the peat burn depth at that point. We followed common protocol to determine burn depths, comparing the base and adventitious roots of intact vegetation indicator species with the location of the residual peat, with the assumption of the pre-fire surface having been flat^33,34^ (Extended Data Fig. 6).

Our field sampling confirmed the presence of peat in 100% of randomly sampled points across vegetation types, and we were able to measure peat burn depths in areas classified as bog, moorlands and heathlands, and forests and woodlands (Extended Data Fig. 6 and Extended Data Table 3). The depth of burn associated was assigned via the values from our sampling described above.

### Quantifying carbon emissions

#### Aboveground emissions

Carbon stocks were apportioned for each 10-m grid cell within the burn perimeter using vegetation type-specific mean carbon stocks according to a static aboveground carbon map of Great Britain^35^. Allocated carbon stocks were capped at 0.17 kgC m^−2^ for aboveground moors and heathland and 0.2 kgC m^−2^ for bog vegetation^36^. This provides a model estimate of carbon density based on the vegetation types and land cover present.

Aboveground emissions were then calculated for each vegetation class within each burned pixel by multiplying the burned fraction, the available biomass carbon, the CC (maximum of 1.0 and minimum of 0.8 for ‘non tree’ vegetation^3^) of different fuel types and the fraction of carbon (assumed to be 50%) in different aboveground fuel types.

#### Belowground emissions

We used two approaches to calculate our own bottom-up estimates for belowground carbon emissions. The first one used burn depths constrained by field data and field-sampled site-specific carbon bulk density values, while the second approach followed the Baker Peat Combustion Model^6^, a UK-wide model that estimates peat combustion based on a soil moisture scaler and uses a national standard carbon bulk density value.

For our site-specific model, we used the 25th, 50th and 75th quantiles of field-measured burn depths within burn severity classes (low, moderate and high) in the bog and ‘moorlands and heathlands’ land cover classes. Given that soil moisture measured via SMAP is at a very coarse scale relative to the finer-scale mosaic of bogs interspersed throughout the landscape, the field sampling is an approach that allows for more localized climatic or topographic influences on burn depths.

We used two field-derived values for carbon bulk density: one for heathlands, which tend to have greater mineral content and higher bulk densities, and one for bogs. For the ‘moorlands and heathlands’ category, the mean carbon bulk density of 58 ± 22 kgC m^−3^ (mean ± s.d.) was determined from seven measurements in a nearby intact heathland, while seven measurements of carbon bulk density in a nearby intact bog provided a mean of 38 ± 27 kgC m^−3^ (mean ± s.d.), used for emissions calculations within the ‘bog’ category. We sampled the upper 0−10 cm of peat at each location using a sharpened steel corer in the heathland site (1 m × ⌀ 4.25 cm) and an Eijkelkamp Russian-auger (0.5 m × 5.2 cm) in the bog site. Carbon content was measured via combustion on an elemental analyser at the University of Cambridge. We assumed a conservative 50% CC for our core estimate but tested the sensitivity to this assumption by recalculating based on 100% CC as well. We describe our sensitivity analyses below.

Carbon emissions from belowground burning were then calculated as follows: carbon emitted from soil organic carbon (SOC) burning = burn depth (based on field-sampled values) × peat carbon bulk density (site-specific values) × peatland fraction burned × CC (0.5 for dry peat).

The second model (based on the Baker Peat Combustion Model) only considers peat combustion in the fraction of each burned area pixel where land cover is denoted as ‘bog’. We estimated peat stocks within bogs using a gridded peat map of Scotland^15^. For the bog pixels, the peat burn depth was estimated using soil moisture extracted from SMAP (top 7 cm of soil). The soil moisture was taken for the day of the fire.

Peat burn depth was calculated as follows: SOC burn depth (cm) = 13.88 × soil dryness − 3.024.

Carbon emissions from belowground burning were then calculated as follows: carbon emitted from SOC burning = burn depth (based on soil moisture) × peat carbon bulk density (for Scotland) × peatland fraction burned × CC (0.5 for dry peat). A nation-specific carbon bulk density for Scotland of 68.64 kg m^−3^ was used for this model. Justification and descriptions of these parameters are included in ref. ^6^.

We also compared our estimates of combined aboveground and belowground emissions with reported values from GFAS. GFAS is based on applying emission coefficients relative to fire radiative power for specific land cover types.

### Sensitivity analyses

To assess the sensitivity of our total emissions estimates to assumptions about peat properties in the belowground combustion model, we performed one-at-a-time scenario tests varying burn depth, peat carbon bulk density and peat CC. Our baseline model used field-measured median burn depth in all vegetation– severity combinations, site-specific carbon bulk density and a conservative CC of 0.5 (ref. ^37^).

#### Bulk density sensitivity

We used measured peat bulk density taken from landscapes adjacent to the Dava Moor burn (referred to as site-specific values). When holding burn depth and CC constant (median burn depth within each burn severity/land cover class; CC of 0.5), replacing site-specific bulk density with the national value used in the Baker Peat Combustion Model increased total fire carbon emissions from 38,500 MgC to 45,300 MgC (+18%).

#### CC sensitivity

CC values used to estimate belowground peat combustion vary widely in the literature, reflecting on differences in fire behaviour, peat condition, formation of pyrogenic carbon and physical factors such as fire-induced compaction of soil. Some previous analyses have assumed complete combustion of peat to the measured burn depth (CC of 1.0)^38,39^, effectively providing an upper-bound estimate of belowground carbon loss under sustained smouldering conditions. By contrast, other studies have emphasized incomplete peat combustion, documenting substantial residual soil carbon following fire and adopting lower CC values to reflect spatially heterogeneous fire severity and moisture conditions^37^. Following this latter approach, we adopt CC of 0.5 as a conservative baseline, while explicitly evaluating the sensitivity of our results to higher CC values used in prior work. Our sensitivity analysis indicates that raising CC from 0.5 to 1.0 leads to an increase in total emissions to 119,000 MgC because this is both higher CC and the 75th percentile of the burn depth measurements.

## Online content

Any methods, additional references, Nature Portfolio reporting summaries, source data, extended data, supplementary information, acknowledgements, peer review information; details of author contributions and competing interests; and statements of data and code availability are available at 10.1038/s41561-026-01994-3.

## Figures and Tables

**Extended Data Table 1:**
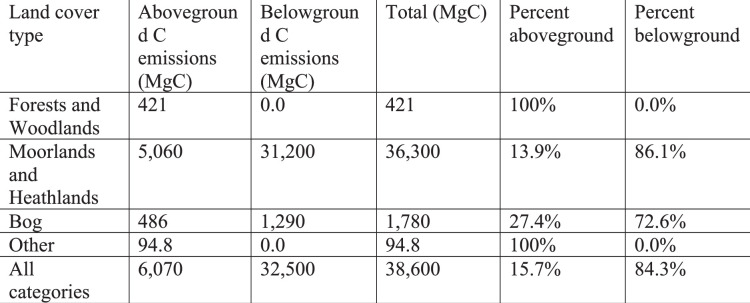
Dava Moor Fire burned area by severity and land cover type

**Extended Data Table 2:**
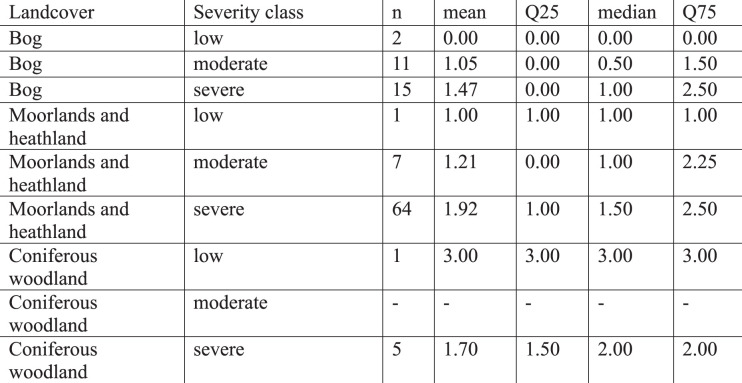
Carbon (C) emissions of the Dava Moor Fire by land cover type, using median field-measured burn depths and field-derived carbon bulk densities

**Extended Data Table 3:**
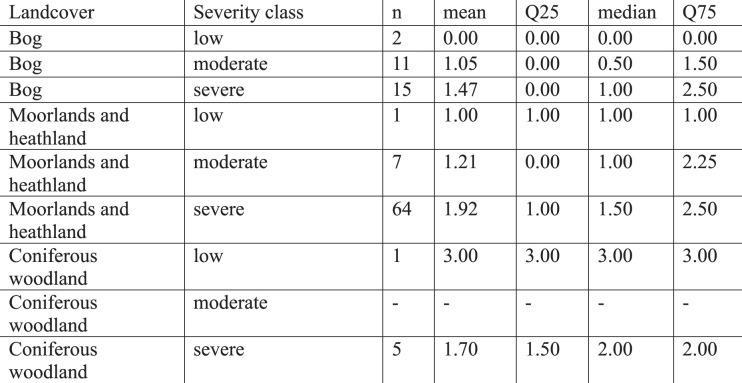
Field-measured peat burn depths (cm) by landcover type and burn severity class (dNBR)

## Data Availability

All data used in this Brief Communication are freely available from the sources cited or via Zenodo at 10.5281/zenodo.19596849 (ref. ^40^).

## Code availability

All code essential for this analysis is available via Zenodo at 10.5281/zenodo.19359438 (ref. ^41^).
